# Uncovering Nonlinear Predictors of Serum Biomarker Uric Acid Using Interpretable Machine Learning in Healthy Men

**DOI:** 10.3390/biomedicines13102469

**Published:** 2025-10-10

**Authors:** Chung-Chi Yang, Min-Chung Shen, Zih-Yin Lai, Jyun-Cheng Ke, Ta-Wei Chu, Yung-Jen Chuang

**Affiliations:** 1 Division of Cardiology, Department of Medicine, Taoyuan Armed Forces General Hospital, Taoyuan 325208, Taiwan; t220979@gmail.com; 2 Cardiovascular Division, Tri-Service General Hospital, National Defense Medical University, Taipei 114202, Taiwan; 3 School of Medicine, National Tsing Hua University, Hsinchu 300044, Taiwan; laiziyin@gmail.com; 4 Institute of Bioinformatics and Structural Biology, National Tsing Hua University, Hsinchu 300044, Taiwan; 5 Division of Rheumatology and Immunology, Taoyuan Armed Forces General Hospital, Taoyuan 325208, Taiwan; airfly100@gmail.com; 6 Department of Obstetrics and Gynecology, Tri-Service General Hospital, National Defense Medical University, Taipei 114202, Taiwan; koyasa0401@gmail.com (J.-C.K.); taweichu@gmail.com (T.-W.C.); 7 MJ Health Research Foundation, Taipei 114066, Taiwan

**Keywords:** uric acid, machine learning, multivariate adaptive regression splines, nonlinear modeling, multiple linear regression

## Abstract

**Background:** Uric acid (UA) is linked to gout, renal dysfunction, and cardiovascular disease. Prior studies often assume linear relationships, potentially oversimplifying physiological complexity. **Methods:** We analyzed data from 5200 healthy Taiwanese men. Demographic, biochemical, lifestyle, and inflammatory variables were assessed using Pearson correlation, multiple linear regression (MLR), and multivariate adaptive regression splines (MARS), an interpretable machine learning method for detecting nonlinear, threshold-based effects. **Results:** Pearson correlation showed broad linear associations, whereas MARS identified fewer but more physiologically meaningful predictors. Waist-to-hip ratio (WHR) had a strong threshold effect, influencing UA only below 0.969. Creatinine showed a nonlinear impact, becoming substantial above 0.97 mg/dL, suggesting a renal threshold within the “normal” range. Calcium and high-sensitivity C-reactive protein (hs-CRP) each displayed inflection points (9.5 mg/dL and 3.38 mg/L, respectively), indicating range-specific effects. Notably, betel nut exposure, nonsignificant in linear models, emerged in MARS as a predictor with a complex, non-binary association with UA metabolism. Predictive performance was comparable (RMSE: 1.6694 for MARS vs. 1.6666 for MLR), but MARS offered superior interpretability by highlighting localized nonlinear effects. **Conclusions:** MARS modeling revealed critical nonlinear, threshold-dependent associations between UA and WHR, creatinine, calcium, hs-CRP, and betel nut exposure, which were not captured by conventional methods. These findings underscore the value of interpretable machine learning in metabolic research and suggest precise thresholds for clinical risk stratification.

## 1. Introduction

Uric acid (UA) is the end product of purine metabolism. About 80% of UA is derived from endogenous metabolism of amino acids and nucleic acids, while the remaining 20% originates from dietary sources rich in purine or nucleic acid proteins [1]. UA is primarily excreted through the kidneys into urine, and excessive intake of purine-rich or nucleic acid-rich foods may elevate serum UA levels and place an additional burden on renal function [1]. According to Taiwan’s Health Promotion Administration, Ministry of Health and Welfare, an average of 750 mg of UA is produced daily, with approximately 500 mg excreted by the kidneys [2]. The remainder is eliminated via bile secretion into the colon and ultimately expelled in feces. Hyperuricemia is defined as serum UA ≥7 mg/dL in men and ≥6 mg/dL in women, respectively, and is associated with increased risks of gout, renal stones, and arthritis [2]. In recent years, UA has attracted increasing clinical attention because of its strong association not only with classical outcomes such as gout and nephrolithiasis, but also with metabolic syndrome and cardiovascular diseases [3,4,5]. Elevated UA levels have been linked to obesity, insulin resistance, hypertension, and dyslipidemia, key components of metabolic syndrome, suggesting that UA plays an integral role in metabolic homeostasis and systemic inflammation [3,4]. Furthermore, accumulating evidence indicates that UA contributes to endothelial dysfunction, oxidative stress, and systemic inflammation, all of which are key mechanisms involved in cardiovascular diseases development [4,5]. One comprehensive review highlighted that elevated UA may affect the activity of enzymes such as nitric oxide synthase, adenosine monophosphate kinase, adenosine monophosphate dehydrogenase, and nicotinamide adenine dinucleotide phosphate, contributing to pathological processes involved in cardiovascular diseases [5]. Therefore, UA is increasingly recognized not only as a biomarker of purine metabolism, but also as an active participant in cardiometabolic disease pathogenesis, reinforcing its clinical significance.

An important and unresolved question is which risk factors interact with UA, and how these relationships may vary across different physiological and biochemical states [6,7]. In particular, understanding whether these associations follow linear or nonlinear patterns can offer critical insights into the underlying mechanisms regulating UA metabolism. Such knowledge can deepen our understanding of physiological phenomena and their implications for disease risk and progression. However, most prior studies have relied on traditional statistical methods, which may fail to capture complex and nonlinear interactions among variables [6,7]. In recent years, the rise of artificial intelligence has brought new analytical tools to biomedical research. Machine learning, in particular, excels in modeling the complexity and nonlinearity of large datasets, outperforming traditional statistical approaches like multiple linear regression (MLR) [8]. To our knowledge, there are few studies that have employed machine learning to explore hyperuricemia risk factors, yet the studies treated UA as a binary variable (presence or absence of hyperuricemia), thus limiting their clinical interpretability and failing to elucidate the full spectrum of UA variation [8,9]. Among various machine learning approaches, multivariate adaptive regression splines (MARS) offers some unique advantages. MARS not only accommodates nonlinear associations but also generates interpretable equations, bridging the gap between black-box models and conventional regression techniques. This makes MARS particularly suitable for clinical applications where transparency and interpretability are essential.

In the present study, we employed MARS to analyze data from healthy Taiwanese men, incorporating demographic, biochemical, lifestyle, and inflammatory markers. Our aim was not simply to build a predictive model, but to leverage an interpretable mathematical formula to understand the underlying relationships between UA and its associated factors, especially their nonlinear patterns and biological implications.

## 2. Materials and Methods

### 2.1. Participant and Study Design

Participant data collection has been previously reported by our group [10]. The present analysis utilized data from the Taiwan MJ Cohort, an ongoing prospective health screening program managed by the MJ Health Screening Centers in Taiwan [11]. The health examinations capture more than 100 biological indicators, including anthropometric parameters, blood biomarkers, and imaging assessments. Participants also complete a self-administered questionnaire covering personal and family medical history, current health conditions, lifestyle habits, physical activity, sleep, and dietary patterns [12].

This study represents a secondary analysis of de-identified data obtained from the MJ Health Clinics. At the time of their health evaluations, all participants provided broad informed consent permitting the use of anonymized data for future research. The dataset is curated and maintained by the MJ Health Research Foundation, and the analyses were conducted under authorization (Authorization Code: MJHRF2023015A). The interpretations and conclusions of this work are solely those of the authors and do not necessarily reflect the views of the Foundation. Additional methodological details are available in the Foundation’s annual technical report [12].

**Figure 1:**
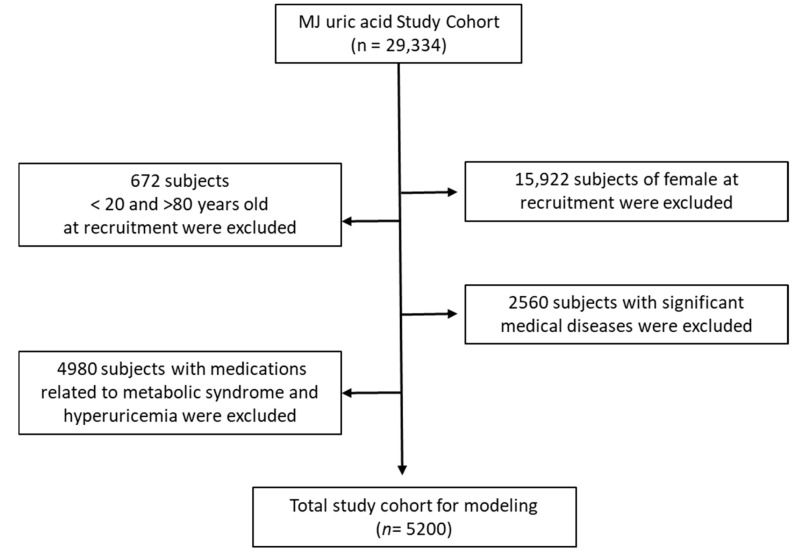
Flowchart of sample selection in the current study.

The study protocol was approved by the Institutional Review Board of Tri-Service General Hospital (IRB No. C202305049). Since no new biological specimens were collected, the study qualified for expedited review and did not require additional informed consent. The study population comprised men aged 20 to 80 years. Participants with a history of cancer or those taking medications for hyperglycemia, hypertension, hyperlipidemia, hyperuricemia, or corticosteroids were excluded. The participant selection process is illustrated in Figure 1.

### 2.2. Laboratory Tests

On the day of the health examination, experienced nursing staff documented each participant’s medical history, including current medication use, and performed a standardized physical examination. Waist circumference was measured horizontally at the natural waist, and body mass index (BMI) was calculated as weight in kilograms divided by height in meters squared. Blood pressure was assessed on the right arm in a seated position using a standard mercury sphygmomanometer to record both systolic and diastolic values. Physical examinations and blood pressure measurements were conducted in accordance with ISO 9001 standards [13] and CAP-accredited laboratory procedures. Abnormal values were re-assessed, and all instruments were regularly calibrated.

Following a 10-h overnight fast, venous blood samples were obtained for biochemical testing. Plasma was separated within one hour of collection and stored at −30 °C until analysis. Fasting plasma glucose (FPG) was measured with the glucose oxidase method (YSI 203 glucose analyzer, Yellow Springs Instruments, Yellow Springs, OH, USA). Total cholesterol and triglycerides were determined using the dry multilayer analytical slide method on the Fuji Dri-Chem 3000 analyzer (Fuji Photo Film, Tokyo, Japan). Serum high-density lipoprotein cholesterol (HDL-C) and low-density lipoprotein cholesterol (LDL-C) were quantified by enzymatic assay following dextran sulfate precipitation. Urinary microalbumin concentrations were measured using turbidimetry on a Beckman Coulter AU5800 biochemical analyzer (Beckman Coulter Inc., Brea, CA, USA).

Demographic data included information on marital status and whether participants had a spouse. Drinking was calculated as the product of total duration of alcohol consumption, drinking frequency, and alcohol concentration. Similarly, the smoking quantity and betel nut exposure were calculated by multiplying the duration, frequency, and quantity (number of cigarettes or betel nuts consumed). Sports was derived from the product of the duration, frequency, and intensity (by type of physical activity. All these parameters were treated as independent variables in the analysis, with serum UA serving as the dependent variable.

### 2.3. Traditional Statistics

An independent t-test was used to compare UA levels between different marital status groups. Since sleep duration and education level are ordinal variables, analysis of variance (ANOVA) was employed to compare UA across their respective categories. Simple correlation was conducted to evaluate the relationships between UA and other continuous variables. All statistical analyses were performed using SPSS software version 19.0 (IBM Inc., Armonk, New York, NY, USA).

### 2.4. Machine Learning Method

In the present study, the MARS technique was employed to analyze the dataset. MARS is a flexible and powerful method for modeling high-dimensional data, utilizing an expansion framework based on product spline basis functions. Importantly, the number of basis functions and their associated characteristics are automatically determined through data-driven processes [14]. Conceptually, MARS is aligned with recursive partitioning methods and is similarly capable of capturing complex, higher-order interactions.

For model construction, the dataset was randomly partitioned into training (80%) and testing (20%) subsets. To optimize the MARS model, hyperparameter tuning was performed within the training subset. Specifically, the training data were further stratified into an internal training set and a validation set. A grid search procedure was implemented across predefined ranges of key hyperparameters, including the maximum number of basis functions and the degree of allowed interactions. Model performance was evaluated using the root mean square error (RMSE) on the validation set, and the configuration yielding the lowest RMSE was retained as the optimal MARS specification. This optimized model was subsequently benchmarked against a conventional MLR model for comparative performance assessment.

Prior to performing the machine learning analysis, all data preprocessing and quality checks were completed. In this study, continuous variables were normalized using Z-score standardization, while skewed biochemical parameters (e.g., triglycerides, uric acid) were log-transformed. Robust scaling was considered for variables with extreme values. Given the very low proportion of missing data, cases with missing values were excluded from the analysis.

During model evaluation, predictive performance was quantified using the independent testing subset that had been withheld from the training process. Given that serum UA was modeled as a continuous outcome, multiple complementary error metrics were computed to provide a robust assessment of model accuracy. Specifically, symmetric mean absolute percentage error (SMAPE), relative absolute error (RAE), root relative squared error (RRSE), and root mean square error (RMSE) were calculated. These metrics collectively capture both absolute and relative deviations between observed and predicted values, as well as sensitivity to large errors. A detailed summary of the evaluation results is presented in Table 1.

To provide a comparative context, the averaged performance metrics of the MARS model were used to benchmark its performance against the MLR model. It is noteworthy that both models, MARS and MLR, were trained and tested on the same dataset, ensuring consistency in evaluation.

For 95% confidence interval, we quantified uncertainty in threshold locations by resampling. When MARS produced hinge terms, thresholds were estimated by bootstrap of the MARS model (B = N; median and 2.5–97.5th percentiles). When MARS did not yield hinges, we estimated per-variable breakpoints using univariate segmented regression with multiple starting values; if that failed, we applied a two-piece linear (hinge) grid search with bootstrap. The method used for each variable is indicated in the table.

All statistical analyses and modeling procedures were conducted using R software version 4.0.5 and RStudio version 1.1.453, with all necessary packages installed. The MARS models were implemented using the “earth” package (version 5.3.3) [15], and hyperparameter tuning was conducted via the “caret” package (version 6.0–94) [16]. The MLR models were developed using the base “stats” package in R (version 4.0.5) with default settings.

## 3. Results

A total of 5200 healthy male participants were included in the final analysis. Their demographic and baseline characteristics are described in detail (Table 2). To explore the relationship between serum UA levels and various demographic, biochemical, and lifestyle variables, Pearson correlation analysis was first conducted. Most variables demonstrated statistically significant associations with UA levels, with the direction of correlation varying across parameters. Notably, LDL-C, plasma phosphorus concentration, alkaline phosphatase, alpha-fetoprotein, carcinoembryonic antigen, homocysteine, fibrinogen, smoking, betel nut exposure, and sports were non-significantly correlated with UA (Table 3). This wide range of significant associations underscores the complexity of the physiological interactions contributing to UA regulation.

To further investigate how social and lifestyle factors influence UA levels, we performed a series of group comparisons. However, only education level showed a significant difference in UA levels, while marital status and sleep duration did not, as determined by t-tests and ANOVA (Table 4). These findings suggest that while metabolic and biochemical markers have measurable correlations with UA, certain social determinants may have limited impact in this healthy male population.

We then compared the performance of two modeling approaches, MLR and MARS, for predicting UA levels. While MARS and MLR showed comparable predictive accuracy, MARS offered substantially greater physiological interpretability by revealing localized, nonlinear effects. (Table 5). This suggests that MARS may offer advantages in handling complex, nonlinear relationships. However, both models explained only a small proportion of the variance (r^2^ ≈ 0.044 for MLR and r^2^ ≈ 0.042 for MARS), underscoring that predictive accuracy was limited despite comparable RMSE values.

We then compared the performance of two modeling approaches, MLR and MARS, for predicting UA levels. Both models performed comparably, with MARS showing a marginally higher RMSE (1.6694 vs. 1.6666) and negligible differences across other metrics (Table 5). This indicates that predictive gains were minimal, and that the principal value of MARS lies in uncovering complex, nonlinear relationships. The MLR equation is expressed as below. Given the standard deviation of UA (1.32), the implied R^2^ was approximately 0.044 for MLR and 0.042 for MARS, indicating that both models explained only about 4% of the variance. Accordingly, we emphasize pattern discovery and physiological interpretation over prediction, and we avoid overstating clinical utility.

UA = −2.047 − 0.016 × Age + 5.159 × WHR − 0.005 × FPG + 0.002 × γ-GT + 0.003 × LDH + 1.195 × creatinine + 0.001 × TG + 0.367 × Ca − 0.011 × betel nut exposure + 0.0005 × Hs-CRP.

In-depth analysis of the final MARS model revealed that only a limited subset of variables contributed substantially to UA prediction. These included waist-to-hip ratio (WHR), creatinine, plasma calcium concentration, high-sensitivity C-reactive protein (Hs-CRP), betel nut exposure (BN), age, γ-glutamyl transferase (γ-GT), FPG, lactate dehydrogenase (LDH), and triglycerides (Table 6). Based on the basis functions in Table 6, the MARS-generated equation for estimating UA is as follows:

UA = 7.187 + 0.0259Max(0, 48 – Age) − 0.012Max(0, Age – 48) − 3.28Max(0, 0.969 − WHR) − 0.009Max(0, 115 – FPG) − 0.01Max(0, FPG – 115) − 0.014Max(0, 49 – γ-GT) −0.005Max(0, 211 – LDH) + 2.882Max(0, creatinine – 0.97) − 0.004Max(0, 207 –TG) + 0.001Max(0, TG – 207) − 0.525Max(0, 9.5 – Ca) + 0.106Max(0, 5 – betel nut exposure) − 0.118Max(0, 3.38 – Hs-CRP) − 0.016Max(0, Hs-CRP – 3.38).

A screenshot is provided in the Supplementary Materials Table S1. By coping and pasting the content in the Word file into Excel and type the related factors into the corresponding Excel cells, the result of the equation will be available at A11.

To enhance clinical interpretability, we compared the MARS-derived thresholds against established clinical cut-offs for metabolic syndrome, diabetes, obesity, and kidney dysfunction (Table 7). These thresholds represent inflection points in the UA–predictor relationship within a healthy cohort, indicating changes in slope rather than disease states. They are not diagnostic cut-offs; their role is hypothesis-generating and requires validation in case–control or longitudinal cohorts, since clinical cut-offs are typically defined by comparing healthy and diseased populations.

For example, the creatinine breakpoint at 0.97 mg/dL reflects the point where UA excretion begins to rise disproportionately, despite lying below the conventional abnormal range for renal impairment. Similarly, the WHR threshold (0.969) highlights an inflection point that may mark a subclinical physiological transition rather than a disease state. However, the 95% confidence interval for this WHR threshold was wide (e.g., 0.92–1.01), overlapping with conventional clinical cutoffs, and the apparent “protective” association below this value should not be interpreted as definitive. This pattern may reflect unmeasured confounding—for example, individuals with lower central adiposity may have different dietary patterns (e.g., lower rice or seafood intake) that influence UA exposure. Moreover, the threshold is data-driven and specific to our cohort; it should not be generalized without external validation. Rather than indicating a clinical intervention point, this finding primarily highlights a potential nonlinearity in the relationship between adiposity and UA metabolism that merits further investigation.

Likewise, the fasting glucose threshold (115 mg/dL) suggests a sensitivity zone within a continuous relationship. These values should therefore be interpreted as physiological markers of sensitivity zones within continuous relationships, not as substitutes for established clinical definitions. Nonetheless, their alignment or divergence from guideline cut-offs suggests that such exploratory thresholds may provide mechanistic insights and inform hypotheses for future longitudinal and case–control investigations.

**Figure 2:**
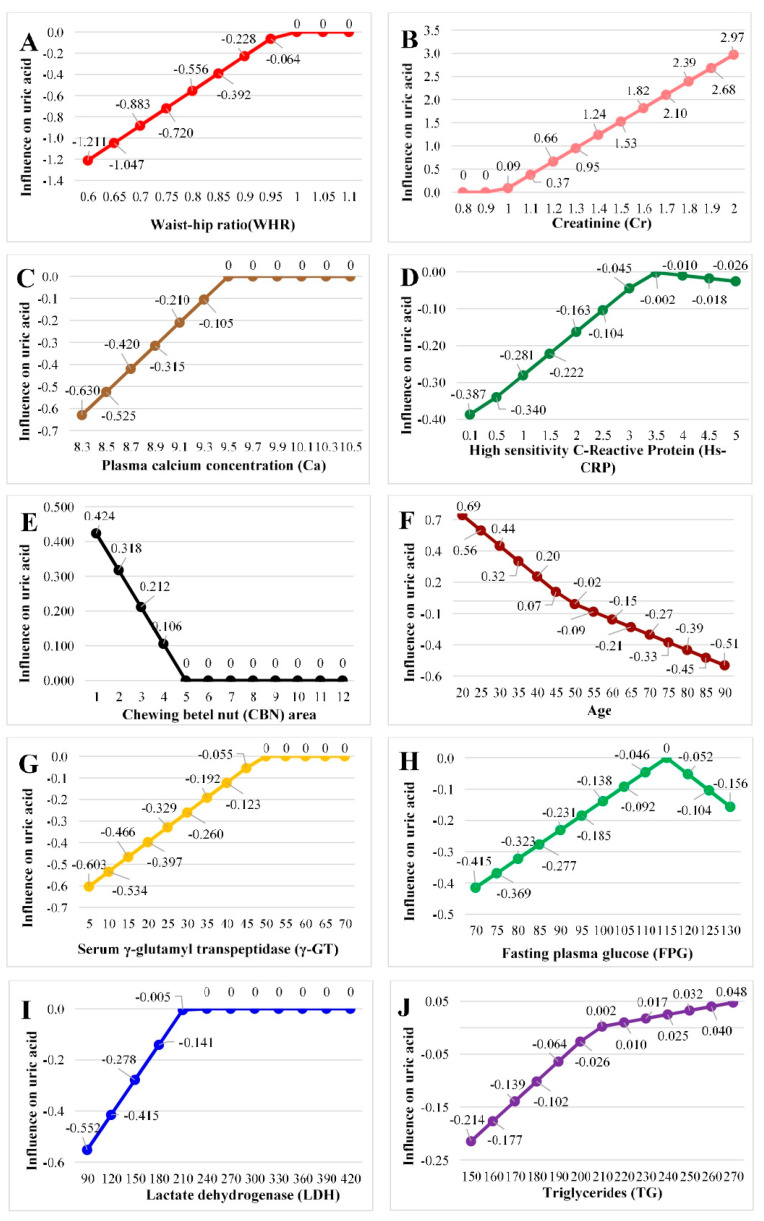
Influence of important variables on uric acid by multivariate adaptive regression splines. Note: (**A**): Waist–hip ratio; (**B**): Creatinine. (**C**): Plasma calcium concentration; (**D**): High-sensitivity C-Reactive Protein; (**E**): Chewing betel nut area; (**F**): Age; (**G**): Serum γ-glutamyl transpeptidase; (**H**): Fasting plasma glucose; (**I**): Lactate dehydrogenase; (**J**): Triglycerides.

Notably, the influence of these variables was not uniformly linear; rather, each variable demonstrated impact on UA only within specific value ranges, as visualized in Figure 2. For example, the effect of WHR on UA was more pronounced below a certain threshold, while creatinine had a sharply positive effect above a specific cutoff. These localized effects highlight the strength of MARS in identifying biologically meaningful, nonlinear associations that would be overlooked by linear models or traditional correlation analysis.

**Figure 3:**
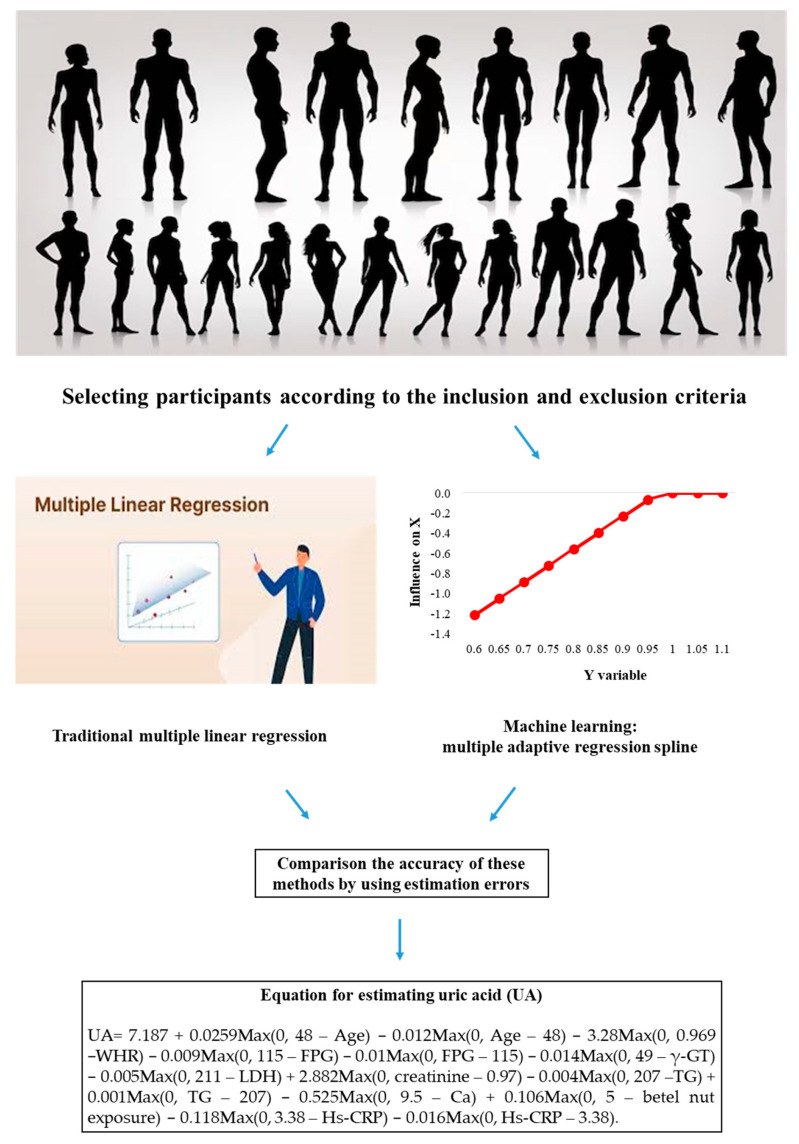
The graphic summary of the present study.

This discrepancy between the broad statistical significance observed in Pearson correlation and the focused, range-specific associations revealed by MARS emphasizes a key methodological insight. While Pearson correlation treats each variable’s effect as constant across its entire range, MARS accommodates complexity and offers a clearer understanding of which variables truly drive UA variability and under what conditions. A schematic overview summarizing the design, analytical workflow, and key findings of the study is provided in Figure 3.

Figure S1 visualizes the estimated turning points in the association between each predictor and UA. Points denote the median threshold, and horizontal bars (and violins when bootstrapped) show the 95% CI for the threshold location; the estimation method used for each variable is listed in Table S2.

Clear, well-localized thresholds (narrow CIs) were observed for γ-GT, TG, LDH, FPG, and age, indicating distinct inflection points in their relationships with UA. In contrast, variables such as Cr, WHR, CRP, calcium, and betel-nut exposure exhibited broader CIs and/or thresholds close to the boundary of their observed ranges, suggesting weaker evidence for a sharp change in slope. Exact point estimates and 95% CIs for all variables are provided in Supplementary Table S2. Notably, the prevalence of individuals beyond each MARS-identified threshold varied widely, offering important context for interpreting their physiological relevance. For instance, creatinine > 0.97 mg/dL affected only 9.8% of participants—yet this small subgroup exhibited a sharp rise in uric acid, highlighting MARS’s sensitivity to detect nonlinear effects even within the conventional “normal” laboratory range. Similarly, fasting plasma glucose > 115 mg/dL, which lies between the thresholds for prediabetes (≥100 mg/dL) and diabetes (≥126 mg/dL), was observed in just 9.2% of the cohort, suggesting that metabolic dysregulation may influence uric acid metabolism earlier than current clinical definitions imply. In contrast, hs-CRP > 3.38 mg/L was present in 36.0% of participants—closely aligning with the established cardiovascular risk cutoff of 3.0 mg/L—and reinforces systemic inflammation as a key driver of uric acid elevation. Calcium < 9.5 mg/dL was remarkably common, affecting 89.4% of the cohort, indicating that even low–normal calcium levels (still within the standard reference range of 8.5–10.5 mg/dL) are physiologically relevant to uric acid regulation. Finally, **betel nut exposure > 5 units was rare (4.1%), yet it emerged as a significant nonlinear predictor, demonstrating MARS’s ability to uncover complex, non-binary associations even in sparse subgroups. Together, these proportions underscore that MARS identifies both common and infrequent—but biologically meaningful—inflection points that linear models overlook.

## 4. Discussion

This study applied a MARS approach to identify and characterize nonlinear associations between serum UA levels and a comprehensive set of demographics, biochemical, lifestyle, and inflammatory factors in a large cohort of healthy Taiwanese men. It should be noted that both MLR and MARS achieved low explanatory power (r^2^ < 0.05), consistent with the weak bivariate correlations. Therefore, while the models provide mechanistic and physiological insight, their utility for individual-level prediction remains limited. While traditional Pearson correlation revealed numerous statistically significant relationships, the MARS model uncovered a more refined and physiologically meaningful set of predictors, including WHR, FPG, creatinine, calcium, Hs-CRP, and betel nut exposure, many of which exhibited threshold-dependent effects not captured by conventional linear models. Our findings highlight the importance of using advanced, interpretable machine learning models to reveal complex, range-specific interactions that may underline metabolic regulation. In particular, the identification of nonlinear breakpoints in variables such as WHR and creatinine underscores the need for precision thresholds in both clinical screening and public health strategies. Moreover, the novel associations found for LDH and betel nut exposure provide new directions for future investigation into metabolic and lifestyle determinants of UA regulation.

It is important to note that both MLR and MARS achieved low coefficients of determination (R^2^ = 0.044 and 0.042, respectively), indicating that the included predictors explain only a small proportion of the total variance in UA concentrations. While MARS did not substantially improve predictive accuracy over MLR (Table 5), its principal contribution lies in uncovering biologically plausible, threshold-dependent relationships that linear models inherently cannot detect. This underscores that the goal of this analysis was explanatory insight, not purely predictive performance.

Although statistically significant associations were observed for several predictors, the overall explanatory power of both models was low (R^2^ < 0.05), consistent with the weak bivariate correlations reported in Table 3. This suggests that the majority of variability in UA levels in this cohort is driven by factors not captured in our dataset—such as unmeasured dietary exposures (e.g., seafood, rice), genetic differences in UA metabolism, or temporal variation in exposure. Consequently, the clinical or public health utility of these models for individual-level prediction is limited, and interpretations should focus on population-level associations rather than predictive accuracy.

The prevalence of obesity has increased dramatically in recent years. According to the World Health Organization, global obesity rates have tripled over the past five decades [17]. Numerous studies have demonstrated a strong association between obesity and elevated UA levels. For example, Li et al. reported that Chinese individuals with high UA levels also had significantly higher triglyceride concentrations [18]. Their multivariate logistic regression model identified a significant association between body mass index and UA (β = 0.202, *p* = 0.039), suggesting that chronic inflammation and oxidative stress associated with obesity may play a mechanistic role [19]. In our study, we selected WHR instead of body mass index as a surrogate marker for obesity. This choice was based on increasing evidence that WHR better reflects central (visceral) adiposity and its metabolic consequences compared to body mass index, which does not distinguish between fat and lean mass or account for fat distribution. WHR emerged as one of the most influential variables in both the Pearson correlation analysis and the MARS model. However, the nature of its association with UA differed substantially between the two methods. Pearson correlation suggested a modest linear relationship between WHR and UA (r = 0.226), implying a uniform increase in UA with increasing WHR. In contrast, the MARS model revealed a pronounced nonlinear, threshold-dependent relationship: WHR significantly influenced UA levels only below a threshold of 0.969, with little to no additional effect observed above this value. Specifically, the basis function Max (0, 0.969–WHR) had the largest absolute coefficient (–3.280) in the MARS model, indicating a sharp decline in UA as WHR increased within the lower range. This implies that the protective effect of low WHR on UA is most prominent below 0.969, and that once WHR exceeds this threshold, its additional impact on UA becomes minimal or flat. This finding is physiologically meaningful. WHR < 0.969 typically represents individuals with relatively low visceral fat accumulation and preserved metabolic homeostasis. In this state, insulin sensitivity remains intact, systemic inflammation is low, and renal UA excretion is likely more efficient. However, as WHR increases beyond this threshold, the metabolic stress from visceral fat accumulation may have already saturated its effect on UA, thereby flattening the curve observed in the MARS model. This threshold phenomenon could not be captured by linear correlation analysis alone and highlights the value of MARS in uncovering nuanced, range-specific relationships.

The second most influential factor associated with serum UA levels in our study was creatinine. A clear positive association was observed between UA and creatinine levels, consistent with findings from previous studies. For instance, Joo et al. demonstrated a dose-dependent relationship between elevated UA and impaired renal function, reporting an adjusted odds ratio of 5.55 (95% CI: 3.27–9.44) for individuals in the lowest quartile of estimated glomerular filtration rate [20]. Several other cross-sectional and longitudinal studies have similarly shown that higher UA levels are associated with progressive decline in renal function [21,22,23,24,25]. The underlying physiological mechanisms linking UA and renal impairment are multifaceted. One key contributor is endothelial dysfunction, which can be induced by elevated serum UA. UA has been shown to inhibit endothelial cell proliferation and reduce the bioavailability of nitric oxide, a critical vasodilator involved in maintaining renal microvascular tone and perfusion [26,27,28]. Reduced NO availability leads to increased vascular resistance and compromised glomerular filtration, thereby contributing to nephron damage. Furthermore, UA may promote oxidative stress and inflammation in renal tissues, exacerbating tubulointerstitial injury and accelerating renal functional decline. As renal function deteriorates, the kidney’s ability to excrete UA diminishes, resulting in further accumulation of UA in the blood. This bi-directional relationship, where UA both contributes to and is affected by renal dysfunction, forms a pathological feedback loop that may explain the strong positive correlation observed in our study. Importantly, the MARS model highlighted a threshold effect, wherein the association between creatinine and UA becomes particularly pronounced above 0.97 mg/dL. This finding suggests that even mild elevations in creatinine, which may still fall within the clinically “normal” range, are associated with disproportionate increases in UA. This reinforces the notion that early renal microvascular changes may already be exerting measurable effects on systemic UA metabolism. Collectively, our findings support a pathophysiological model in which elevated UA not only reflects declining renal clearance but may also act as a contributing factor in the progression of renal dysfunction through mechanisms involving endothelial injury, oxidative stress, and altered hemodynamics.

Calcium plays a vital role in a wide range of cellular functions, including muscle contraction, hormone secretion, nerve conduction, and the activation of numerous enzymes [29]. Both calcium and UA are well-established contributors to the formation of urinary tract stones [30]. However, the relationship between serum UA and calcium levels remains controversial, with prior studies reporting inconsistent or conflicting results [31,32,33,34,35]. In our study, we observed a significant and independent positive correlation between serum UA and calcium levels, as identified by both Pearson correlation and the MARS model. One plausible explanation for this association is the involvement of chronic inflammation. Previous studies have shown that elevated UA is associated with increased levels of pro-inflammatory cytokines such as interleukin-6 and tumor necrosis factor-alpha [36,37,38]. Similarly, hypercalcemia has been linked to heightened inflammatory states, including elevations in C-reactive protein and interleukin-6 levels [39,40]. These parallel findings support the hypothesis that inflammation may act as a common underlying mechanism linking elevated serum levels of UA and calcium. The simultaneous elevation of these markers may thus reflect a shared pathophysiological response to systemic inflammatory burden. Further supporting this hypothesis is the role of Hs-CRP, which emerged as the fourth most influential variable in our MARS model. Hs-CRP is a well-established marker of chronic low-grade inflammation and has been recognized since the 1990s as an independent predictor of cardiovascular events, confirmed by over 25 large-scale epidemiological studies [41]. In parallel, UA has been increasingly recognized not only as a marker of cardiovascular risk but also as a potential pro-inflammatory mediator [42]. For instance, Spiga et al. stratified UA levels into quartiles among 2731 non-diabetic individuals and reported that Hs-CRP levels were significantly higher in the highest UA quartile [42]. Our results are consistent with this literature, further reinforcing the interconnection between elevated UA and systemic inflammation, as reflected by Hs-CRP. In addition to metabolic and inflammatory factors, lifestyle behaviors may also play a role in modulating UA levels. Betel nut exposure, a culturally prevalent practice in Southeast Asia, has been associated with a range of adverse health outcomes. For example, Huang et al. reported a significant association between betel nut exposure and an increased risk of metabolic syndrome [43], while other studies have suggested a possible role in promoting kidney stone formation [44]. Interestingly, a study by Tai et al. found an inverse association between betel nut use and hyperuricemia, with an odds ratio of 0.75 (95% CI: 0.66–0.84) [45]. However, their findings were based on logistic regression, which treats hyperuricemia as a binary outcome, thus limiting interpretation to the presence or absence of disease. In contrast, our use of the MARS model enabled the assessment of continuous, nonlinear associations between betel nut exposure and serum UA levels. This analytical approach revealed a nuanced dose–response relationship, suggesting that betel nut exposure may influence UA metabolism in a non-uniform manner. This novel finding adds depth to the current understanding of lifestyle and UA interactions and highlights the utility of MARS in uncovering complex, range-specific patterns that are not easily captured by traditional models.

The remaining four variables in the MARS model, age, γ-GT, FPG, and LDH, had comparatively smaller coefficients, indicating more modest contributions to serum UA levels. Nonetheless, their associations offer additional physiological insights. Age demonstrated a positive, albeit mild, association with UA. This aligns with findings from Kuzuya et al., who reported a positive longitudinal relationship between age and UA levels in a large cohort of 80,506 individuals of both sexes [46]. The age-related increase in UA may reflect cumulative oxidative stress, decreased renal clearance, or age-associated changes in purine metabolism. Interestingly, our study revealed a positive association between γ-GT and UA, contrary to several earlier studies that reported a negative relationship. Those studies were often conducted in disease-specific populations, such as individuals with diabetes [47], alcohol-related liver disease [48], or patients with metabolic syndrome [49,50]. In contrast, our analysis was performed in a healthy population, suggesting that γ-GT may correlate with UA even in the absence of overt disease. Given γ-GT’s role in glutathione metabolism and oxidative stress response, it is plausible that low-grade oxidative processes contribute to UA elevation even in subclinical states. For fasting plasma glucose, previous research has largely indicated a positive association with UA. However, our analysis supports a modest inverse relationship, in line with recent interventional evidence. Notably, a meta-analysis by Chen et al. involving four clinical trials and 314 patients found that treatment with allopurinol led to significant reductions in FPG (weighted mean difference: −0.61 mmol/L, 95% CI: −0.93 to −0.28) [51]. This observation suggests a potential bi-directional interaction between glucose metabolism and UA, possibly mediated through insulin resistance or oxidative stress pathways. Regarding LDH, existing literature linking this enzyme to UA has been mostly limited to pathological contexts such as preeclampsia [9,52]. LDH, a key enzyme in anaerobic glycolysis, may reflect underlying subclinical tissue turnover or low-grade inflammation, both of which could contribute to increased UA production. This novel finding positions LDH as a potentially underrecognized biomarker in UA regulation, meriting further investigation. Finally, triglycerides were identified as the least influential factor in the MARS model. Although a positive correlation between triglycerides and UA has been widely reported, such as in the small-scale study by Tariq et al. [53], the strength of this association was relatively weak in our analysis. One plausible explanation is shared dietary confounding, particularly high fructose intake. Fructose is known to simultaneously stimulate hepatic UA synthesis and triglyceride-rich lipoprotein production [54,55]. Thus, while triglycerides remain a relevant biomarker in hyperuricemia, its direct mechanistic link to UA may be secondary to underlying metabolic drivers such as diet composition, especially fructose consumption.

This study has several limitations that should be acknowledged. First, it employed a cross-sectional design, which inherently limits the ability to infer causal relationships between variables. Unlike longitudinal studies, this design cannot determine temporal sequences or directionality of associations. Second, the study population consisted exclusively of individuals from a single ethnic group, which may limit the generalizability of the findings. Caution is therefore warranted when extrapolating these results to other ethnic or demographic populations, as genetic, environmental, and cultural factors may influence uric acid metabolism and its associated risk factors. Lastly, it is noteworthy that while uric acid functions as a potent antioxidant in plasma under physiological conditions [1], this protective role is likely negated—or even reversed—in the context of renal dysfunction. As our MARS model highlights, even mild creatinine elevations (0.97 mg/dL), below conventional renal impairment thresholds, are associated with disproportionate UA increases. This suggests that once renal excretory capacity begins to falter, UA transitions from an antioxidant to a pro-oxidant and pro-inflammatory mediator within tissues—particularly in the kidney and vasculature [26,28]. Thus, the clinical implications of elevated UA must be interpreted in the context of renal function: what may be protective in a healthy individual could become pathogenic in early renal stress—a nuance captured by our threshold-based modeling.

## 5. Conclusions

This study leveraged the MARS model to uncover nonlinear, range-specific predictors of serum uric acid in healthy men, an advancement over traditional linear methods. Key variables such as WHR, creatinine, and hs-CRP showed threshold-dependent effects, offering novel physiological insights. Our findings highlight the model’s potential to enhance metabolic risk assessment through interpretable machine learning.

## Figures and Tables

**Table 1:** Equations for calculating performance metrics.

Metric	Description	Calculation
SMAPE	Symmetric Mean Absolute Percentage Error	$SMAPE=\frac{1}{n}\overset{n}{\underset{i=1}{\sum }}\frac{\left|y_{i}-{\hat{y}}_{i}\right|}{\left(\left|y_{i}\right|+\left|{\hat{y}}_{i}\right|\right)/2}$
RAE	Relative absolute error	$RAE=\sqrt{\frac{\sum_{i=1}^{n}{\left(y_{i}-{\hat{y}}_{i}\right)}^{2}}{\sum_{i=1}^{n}{\left(y_{i}\right)}^{2}}}$
RRSE	Root relative squared error	$RRSE=\sqrt{\frac{\sum_{i=1}^{n}{\left(y_{i}-{\hat{y}}_{i}\right)}^{2}}{\sum_{i=1}^{n}{\left(y_{i}-\bar{y_{\dot{i}}}\right)}^{2}}}$
RMSE	Root mean squared error	$RMSE=\sqrt{\frac{1}{n}\overset{n}{\underset{i=1}{\sum }}{\left(y_{i}-{\hat{y}}_{i}\right)}^{2}}$

${\hat{y}}_{i}$ and $y_{i}$ represent predicted and actual values, respectively; n stands for the number of instances.

**Table 2:** Demographic data of participants.

Variables	Unit	Mean ± SD
Age	year	42.05 ± 11.48
Waist–hip ratio	waist/hip circumference	0.86 ± 0.06
Leukocyte	×10^3^/μL	6.16 ± 1.61
Hemoglobin	×10^6^/μL	15.29 ± 1.07
Platelets	×10^3^/μL	227.05 ± 47.99
Fasting plasma glucose	mg/dL	103.40 ± 18.70
Systolic blood pressure	mmHg	119.54 ± 15.24
Diastolic blood pressure	mmHg	76.86 ± 10.46
Total bilirubin	mg/dL	1.06 ± 0.41
Albumin	mg/dL	4.47 ± 0.20
Globulin	g/dL	3.02 ± 0.32
Alkaline Phosphatase	IU/L	62.41 ± 20.85
Glutamic oxaloacetic transaminase	IU/L	25.81 ± 12.23
Glutamic pyruvic transaminase	IU/L	34.87 ± 25.73
γ-glutamyl transpeptidase	IU/L	38.38 ± 50.17
Lactate dehydrogenase	mg/dL	162.52 ± 34.13
Creatinine	mg/dL	1.08 ± 0.18
Triglycerides	mg/dL	133.31 ± 131.20
High density lipoprotein cholesterol	mg/dL	52.63 ± 11.74
Low density lipoprotein cholesterol	mg/dL	123.38 ± 33.32
Plasma calcium concentration	mg/dL	9.49 ± 0.36
Plasma phosphate concentration	mg/dL	3.55 ± 0.48
Alpha-fetoprotein	ng/mL	2.93 ± 1.96
Carcinoembryonic antigen	ng/mL	3.47 ± 96.08
Thyroid stimulating hormone	μIU/mL	1.64 ± 1.38
C reactive protein	mg/dL	0.23 ± 0.45
Bone mass density	T-score	0.51 ± 1.22
Alcohol consumption	-	7.39 ± 18.96
Smoking	-	9.59 ± 20.65
Betel nut exposure	-	0.97 ± 7.41
Sports	-	7.62 ± 8.97
Homocysteine	μmol/L	10.46 ± 4.36
High sensitivity C-Reactive Protein	mg/L	2.08 ± 4.55
Ferritin	ng/mL	252.82 ± 166.95
Fibrinogen	mg/dL	274.86 ± 58.48
Uric acid	mg/dL	6.58 ± 1.32
Marriage status	*n* (%)	
Single	1600 (30.77%)	
With spouse	3600 (69.23%)	
Education level	n (%)	
Illiterate	7 (0.13%)	
Elementary school	97 (1.87%)	
Junior high school (vocational)	210 (4.04%)	
High school	1049 (20.17%)	
Junior college	993 (19.10%)	
University	1900 (36.54%)	
Graduate school or above	944 (18.15%)	
Sleep hours	n (%)	
<4 h/day	82 (1.58%)	
4–6 h/day	1276 (24.54%)	
6–7 h/day	2528 (48.62%)	
7–8 h/day	1114 (21.42%)	
8–9 h/day	166 (3.19%)	
>9 h/day	34 (0.65%)	

**Table 3:** Pearson’s r between uric acid and demographic, biochemistry and lifestyle parameters.

Variable	r	Variable	r	Variable	r	Variable	r
Age	−0.1057 ***	Leukocyte	0.1311 ***	GOT	0.1694 ***	Creatinine	0.1708 ***
Alcohol	0.0764 ***	Hemoglobin	0.1095 ***	GPT	0.2222 ***	CRP	0.027 *
Smoking	0.0151	Platelets	0.1064 ***	Albumin	0.146 ***	Hs-CRP	0.0445 **
Betel nut	−0.0164	TG	0.1924 ***	Globulin	0.1126 ***	CEA	0.0061
Sports	−0.0029	HDL-C	−0.1461 ***	TBIL	−0.0497 ***	TSH	0.0438 **
WHR	0.2256 ***	LDL-C	0.1649	γ-GT	0.1781 ***	Hcys	0.0766
BMD	0.1412 ***	FPG	−0.0309 *	LDH	0.1366 ***	Ferritin	0.1288 ***
SBP	0.1458 ***	Ca	0.1666 ***	ALP	0.0309 *	Fibrinogen	0.0593
DBP	0.1383 ***	P	0.0695	AFP	0.0055		

Note: WHR: waist–hip ratio; BMD: bone mass density; SBP: systolic blood pressure; DBP: diastolic blood pressure; TG: triglycerides; HDL-C: high-density lipoprotein cholesterol; LDL-C: low density lipoprotein cholesterol; FPG: fasting plasma glucose; Ca: plasma calcium concentration; P: plasma phosphate concentration; GOT: serum glutamic oxaloacetic transaminase; GPT: serum glutamic pyruvic transaminase; TBIL: total bilirubin; γ-GT: serum γ-glutamyl transpeptidase; LDH: lactate dehydrogenase; ALP: alkaline Phosphatase; AFP: alpha-fetoprotein; CRP: C-reactive protein; Hs-CRP: high sensitivity C-reactive protein; CEA: carcinoembryonic antigen; TSH: thyroid stimulating hormone; Hcys: homocysteine.*: *p* < 0.05; **: *p* < 0.01; ***: *p* < 0.001.

**Table 4:** T-test between uric acid and marital status, analysis of variance between uric acid and education level, and sleep hours.

Variables	Uric Acid (mg/dL)	*p* Value
Marriage status		
Single	6.62 ± 1.33	0.075
With spouse	6.56 ± 1.31	
Education level		
Illiterate	7.53 ± 1.61	0.005
Elementary school	6.21 ± 1.11	
Junior high school (vocational)	6.60 ± 1.41	
High school	6.62 ± 1.40	
Junior college	6.59 ± 1.33	
University	6.58 ± 1.27	
Graduate school or above	6.53 ± 1.28	
Sleep hours		
<4 h/day	6.62 ± 1.44	0.936
4–6 h/day	6.59 ± 1.32	
6–7 h/day	6.56 ± 1.31	
7–8 h/day	6.59 ± 1.32	
8–9 h/day	6.61 ± 1.35	

**Table 5:** The average performance of multiple linear regression and multivariate adaptive regression splines.

Methods	SMAPE	RAE	RRSE	RMSE
MLR	0.1797	1.1250	1.2542	1.6666
MARS	0.1789	1.1199	1.2563	1.6694

Note: MLR: multiple linear regression, MARS: multivariate adaptive regression splines.

**Table 6:** List of basis function *B*_i_ of the MARS model and their coefficients, a_i_.

	Definition	a1
Intercept		7.187
*B*1	Max(0, 48–Age)	0.025
*B*2	Max(0, Age–48)	−0.012
*B*3	Max(0, 0.969–WHR)	−3.280
*B*4	Max(0, 115–FPG)	−0.009
*B*5	Max(0, FPG–115)	−0.010
*B*6	Max(0, 49–γ-GT)	−0.014
*B*7	Max(0, 211–LDH)	−0.005
*B*8	Max(0, Cr–0.97)	2.882
*B*9	Max(0, 207–TG)	−0.004
*B*10	Max(0, TG–207)	0.001
*B*11	Max(0, 9.5–Ca)	−0.525
*B*12	Max(0, 5–BN)	0.106
*B*13	Max(0, 3.38–Hs-CRP)	−0.118
*B*14	Max(0, Hs-CRP–3.38)	−0.016

Note: WHR: Waist–hip ratio; FPG: Fasting plasma glucose; γ-GT: Serum γ-glutamyl transpeptidase; LDH: Lactate dehydrogenase; Cr: Creatinine; TG: Triglycerides; Ca: Plasma calcium concentration; BN: betel nut exposure; Hs-CRP: High sensitivity C-Reactive Protein.

**Table 7:** Comparison of MARS-Identified Thresholds for Uric Acid Predictors with Established Clinical Cut-offs for Metabolic and Renal Disorders.

Variable	MARS Threshold (Present Study)	Clinical Threshold for Metabolic Syndrome/Obesity/Diabetes/Kidney Disease	Source/Guideline	Interpretation/Clinical Relevance
WHR	<0.969 (impact zone)	≥0.90 (abdominal obesity, men)	IDF 2006; WHO 2008	MARS identifies a protective” effect below 0.969—still above the obesity threshold (0.90)
Creatinine	>0.97 mg/dL	>1.1–1.3 mg/dL (mild renal impairment, men)	KDIGO 2012; Lab reference ranges	MARS detects UA elevation before creatinine reaches clinical “abnormal” range—supports early renal stress even within “normal” labs.
hs-CRP	>3.38 mg/L	>3.0 mg/L (high cardiovascular risk)	AHA/CDC 2003	MARS threshold aligns closely with established CV risk stratification—reinforces inflammation as key UA driver.
Fasting Glucose	>115 mg/dL	≥100 mg/dL (prediabetes); ≥126 mg/dL (diabetes)	ADA 2023	MARS identifies nonlinear effect starting at 115 mg/dL —between prediabetes and diabetes thresholds, suggesting glucose dysregulation impacts UA before frank diabetes.
Calcium	<9.5 mg/dL	Normal range: 8.5–10.5 mg/dL; no direct MetS threshold	Lab reference ranges	MARS suggests low–normal calcium (still within “normal”) is associated with higher UA—possibly reflecting subclinical inflammation or bone turnover.
Triglycerides	>207 mg/dL	≥150 mg/dL (MetS criterion)	NCEP ATP III 2001	MARS effect activates well above MetS threshold— suggests UA is more sensitive to severe hypertriglyceridemia than mild elevations.
γ-GT	<49 IU/L	Normal: <55–65 IU/L (lab-dependent); no direct MetS threshold	Lab reference ranges	MARS identifies effect in low–normal range— may reflect hepatic oxidative stress before enzyme elevation becomes clinically apparent.

Note: IDF = International Diabetes Federation; WHO = World Health Organization; KDIGO = Kidney Disease: Improving Global Outcomes; AHA = American Heart Association; CDC = Centers for Disease Control; ADA = American Diabetes Association; NCEP ATP III = National Cholesterol Education Program Adult Treatment Panel III. All clinical thresholds are for adult males unless otherwise specified. “MARS Threshold” indicates the value at which the relationship with UA changes significantly per basis functions in Table 6.

## Data Availability

Data available on request due to privacy/ethical restrictions. This study used secondary databases for analysis. The source of the database was from the MJ Health Research Foundation.

## Supplementary Materials

The following supporting information can be downloaded at: https://www.mdpi.com/article/10.3390/biomedicines13102469/s1, Figure S1: Threshold locations with 95% confidence intervals for predictors of serum uric acid; Table S1: The details of equation which could be copied to Excel file for estimating UA; Table S2: Estimated thresholds and 95% confidence intervals for predictors of serum uric acid.
