# Supplementary material for: Uncovering Nonlinear Predictors of Serum Biomarker Uric Acid Using Interpretable Machine Learning in Healthy Men
Source: Biomedicines. 2025 Oct 10;13(10):2469. doi: 10.3390/biomedicines13102469 (PMC12561602; doi:10.3390/biomedicines13102469)
Supplement: Supplementary file 1 [file biomedicines-13-02469-s001.zip › biomedicines-3873007-supplementary.pdf]

Table S1. The details of equation which could be copied to Excel file for estimating UA.

	A	B	C
1	Type Age	=MAX(0, 48 - Age)	=0.025*B1
2		=MAX(0, Age - 48)	=-0.012*B2
3	Type WHR	=MAX(0, 0.969 - WHR)	=-3.280*B3
4	Type FPG	=MAX(0, 115 - FPG)	=-0.009*B4
5		=MAX(0, FPG - 115)	=-0.010*B5
6	Type γ-GT	=MAX(0, 49 - γ-GT)	=-0.014*B6
7	Type LDH	=MAX(0, 211 - LDH)	=-0.005*B7
8	Type Cr	=MAX(0, Cr - 0.97)	=2.882*B8
9	Type TG	=MAX(0, 207 - TG)	=-0.004*B9
10		=MAX(0, TG - 207)	=0.001*B10
11	Type Ca	=MAX(0, 9.5 - Ca)	=-0.525*B11
12	Type BN	=MAX(0, 5 - BN)	=0.106*B12
13	Type Hs-CRP	=MAX(0, 3.38 - Hs-CRP)	=-0.118*B13
14		=MAX(0, Hs-CRP - 3.38)	=-0.016*B14
15			
16			
17	Intercept		7.187
18	UA		=7.187 + SUM(C1:C14)

Table S2. Estimated thresholds and 95% confidence intervals for predictors of serum uric acid

Variable	Median	CI-low	CI-high	N non-missing	Side reported	N beyond	Pct beyond	Pct beyond CI	N other	Pct other	Pct other CI
Age	36.82	33.29	41.235	6758	above	4388	64.9	63.8-66.1	2370	35.1	33.9-36.2
Betel nut	0	0	0	6593	above	269	4.1	3.6-4.6	6324	95.9	95.4-96.4
Calcium	10	9.1	10	6462	below	5777	89.4	88.6-90.1	685	10.6	9.9-11.4
FPG	114	110.76	114	6763	above	623	9.2	8.5-9.9	6140	90.8	90.1-91.5
WHR	0.92	0.792	0.937	6762	above	988	14.6	13.8-15.5	5774	85.4	84.5-86.2
CRP	1.53	1.31	2.411	6763	above	2435	36	34.9-37.2	4328	64	62.8-65.1
TG	207.76	162.24	228	6763	above	888	13.1	12.3-14.0	5875	86.9	86.0-87.7
γ-GT	46.76	40.41	59.907	6760	above	1435	21.2	20.3-22.2	5325	78.8	77.8-79.7
LDH	196	183.82	196	6713	above	672	10	9.3-10.8	6041	90	89.2-90.7
Creatinine	1.23	0.942	1.23	6763	above	660	9.8	9.1-10.5	6103	90.2	89.5-90.9

N non-missing is the number of participants with available values for that variable and is used as the denominator for proportions. Side reported indicates which side of the threshold was summarized (default above the threshold; below for markers where lower values are clinically pertinent, e.g., calcium). Pct beyond is the proportion of participants on the reported side with 95% confidence intervals (CIs) computed using the Wilson score method; complementary counts and percentages (N other, Pct other) are shown for completeness. Thresholds are model-derived inflection points in the predictor–uric acid association and should not be interpreted as diagnostic cut-offs. Units: Age (years); Betel nut (ordinal exposure score); Calcium (mg/dL); FPG (mg/dL); WHR (unitless); CRP (mg/L); TG (mg/dL); γ-GT (U/L); LDH (U/L); Creatinine (mg/dL). Values are rounded; minor discrepancies may occur due to rounding.

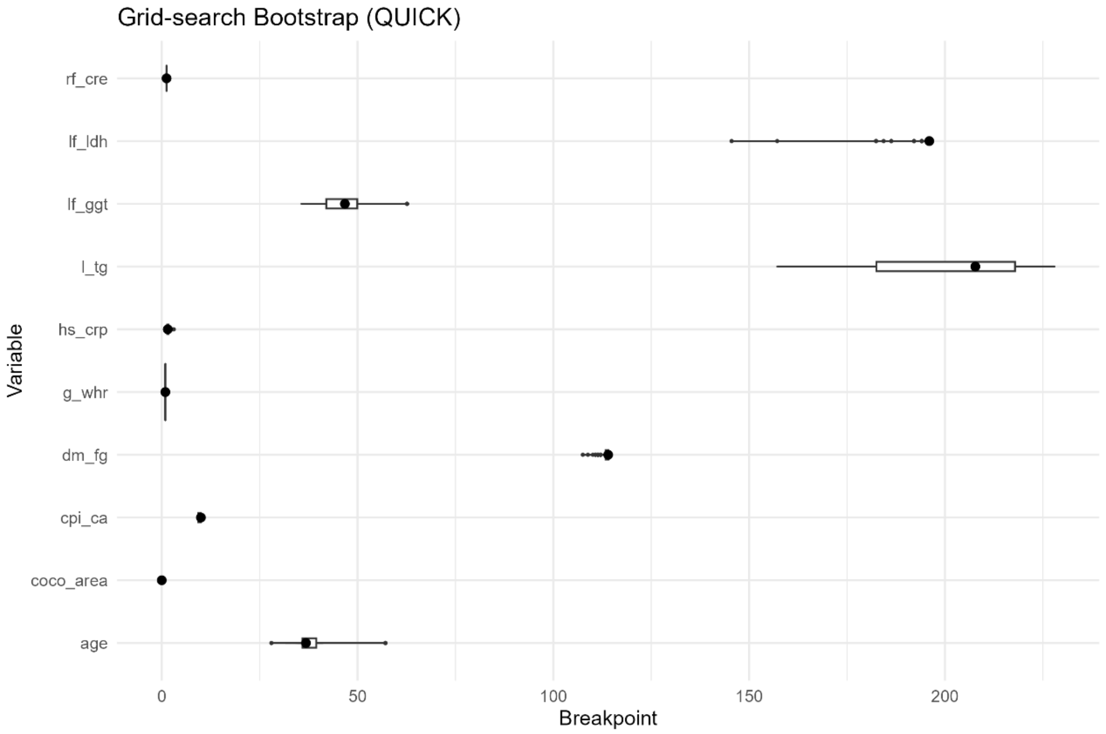

Figure S1. Threshold locations with 95% confidence intervals for predictors of serum uric acid. Points show median threshold estimates; horizontal bars show 95% CIs. Where bootstrap was available (MARS hinges or grid-search), violins/boxes depict the bootstrap sampling distribution of the threshold; where only segmented estimates were available, the point with error bar is shown.
